# Relevance of functional imaging in dental implantology

**DOI:** 10.4317/jced.54816

**Published:** 2018-10-01

**Authors:** Vincent Benouaich, Anne Hitzel, Serge Armand

**Affiliations:** 1 Doctor of Dental Surgery, Department of Odontology, Toulouse University Hospital, France; 2 Doctor of Medicine, Department of Nuclear Medicine, Toulouse University Hospital, France; 3 Doctor of Dental Surgery, University Professor, Department of Odontology, Toulouse University Hospital, France

## Abstract

**Background:**

Despite it is widely used in many medicine fields, the use of functional imaging to examine dental implants has not been reported in the literature. This work aimed to evaluate the relevance of functional medical imaging in oral implantology.

**Material and Methods:**

This single-center observational study was conducted for 6 months at the Toulouse University Hospital, France. All patients who underwent positron emission tomography with 18-fluorodeoxyglucose integrated with X-ray computed tomography (FDG PET/CT) and had dental implants were included. Metabolic activity of the peri-implant tissues was assessed qualitatively and quantitatively jointly by a nuclear physician and a dental surgeon.

**Results:**

In 31 patients (121 implants), peri-implant metabolic activity was normal. In 3 patients (4 implants), localized peri-implant hypermetabolism was observed. In all the patients presenting abnormal peri-implant activity, the implants with normal activity were clinicaly and radiogicaly normal, whereas those with hypermetabolism presented peri-implantitis.

**Conclusions:**

This study assess of the relevance of FDG PET/CT in oral implantology. It shows a link between peri-implant hypermetabolism and peri-implantitis. Therefore, FDG PET/CT could become a new tool for the assessment of peri-implant diseases.

** Key words:**Dental implantation, dental implants, peri-implantitis, diagnostic imaging , imaging, three-dimensional, imaging processing, computer-assisted.

## Introduction

Since the insertion of the first dental implants in the 1960s, the field of implantology has expanded widely (1,2). In parallel with the development of implant techniques, medical imaging has been modernized with digital intraoral radiography, digital panoramic radiography, X-ray computed tomography (CT), and the wider application of cone-beam computed tomography (CBCT) (3). Imaging is of fundamental relevance, not only for the planning and realization of dental implant projects, but also for implant monitoring (4-6). One of the aims of such monitoring is to characterize and understand the etiology of implant loss to improve its treatment (7).

Peri-implantitis is a complex pathology that has infectious and inflammatory etiologies (8-10). It is the leading cause of implant loss (11). Its diagnosis is based on clinical (gingival recession, peri-implant probing, bleeding, mobility) and radiological (bone loss between assessments) aspects (1). Although routinely used radiological assessments enable the acquisition of very accurate morphological data, they provide only a retrospective view of the lesions caused by peri-implantitis, with no functional or physiological information.

Functional medical imaging provides physiological and physiopathological information. It has allowed much progress in all areas of modern medicine, in research and in clinical practice. The purpose of this work was to evaluate the relevance of functional imaging in implantology. The goals were to describe normal and pathological image features, and to consider the establishment of correlations at the anatomical and clinical levels.

## Material and Methods

In order to evaluate the relevance of functional imaging in implantology, a single-center observational study was conducted at the Toulouse University Hospital, France. All patients who underwent positron emission tomography with 18-fluorodeoxyglucose integrated with X-ray computed tomography (FDG PET/CT) between 15 September 2016 and 16 February 2017 and had dental implants were included.

FDG PET examinations of the whole body above the knees were performed according to the clinic’s protocol after the patient had rested for 2 h and fasted for 4 h. A peripheral venous catheter was placed. Glycemic control was achieved, followed by an intravenous bolus injection of 18FDG at a dosage of 2.5 mBq/kg body weight. The patient then rested for 60 min in a quiet space. The patient was then set lying on his back and immobilized in a comfortable position for FDG PET/CT examination.

The PET camera used was installed in 2008 at the Toulouse University Hospital in Purpan and consisted of a Biograph 6 model (Siemens, Knoxville, TN, USA) coupled with full-body CT equipment. The PET component was composed of 32448 lutetium oxyorthosilicate crystals, enabling acquisition of 109 sections in a physical axial field of view of 21.6 cm. The measurements needed for auto-attenuation correction were acquired using the CT scanner of the Biograph 6. This device has software to correct the partial volume effect, which yields post-correction spatial resolutions close to 2.5 mm. The images were subjected to iterative reconstruction (14 subsets, 4 iterations) with Gaussian filtering (full-width half maximum = 5 mm), yielding 74 sections of 128 × 128 pixels.

Peri-implant imaging was performed jointly by a nuclear physician and a dental surgeon. To perform the quantitative measurements, the occlusal plane was chosen as a reference plane to position the acquisition volume. To measure the standardized uptake value (SUV), a section parallel to the occlusal plane passing through the neck of each implant was selected. For peri-implant SUV measurement, a region of interest (ROI) was defined by 3 discs of 2-mm diameter centered in the peri-implant area. A control ROI was defined using a 5-mm diameter disc located on the cancellous bone of the ramus. The maximum standardized uptake value (SUVmax) was used to quantify the peri-implant SUV. For the control SUV, the mean standardized uptake value (SUVmean) was measured.

Quantitative peri-implant hypermetabolism was defined as a SUVmax > 5. Qualitative peri-implant hypermetabolism was visualy assessed comparing the peril-implant ROI to the control ROI. Statistical analysis (mean, standard deviation and student test) was performed using Microsoft Excell 2007 (Microsoft, Redmond, Washington).

For all cases showing qualitative and quantitative hypermetabolism, clinical and paraclinical oral assessments were performed to establish associations between functional abnormalities and implant pathologies.

This study was approved by the ethics committee of our institution, all data has been anonymized, and the rights of patients were respected at every step of the study. This human observational study conforms to the STROBE statement. 

## Results

-Sample characteristics

Thirty-two patients [17 (53%) women, 15 (47%) men] with a mean age of 63.3 ± 9.29 years were included in this study. These patients had a total of 126 implants, with an average of 3.94 (range, 1–16) implants per patient.

In all cases, FDG PET/CT scans were performed for the detection of neoplastic pathologies, including initial assessments of extent and monitoring for recurrence and of active lesions. All dental implants had been inserted >6 months before patients underwent FDG PET/CT.

-Normal peri-implant tissues

Quantitative and qualitative normal SUV were found in 122 out of 126 implants (96.8%). On normal images, the peri-implant metabolic activity was visually identical to the activity of surrounding bone and periodontal tissue. The mean peri-implant SUVmax was 1.43 ± 1.67 and the control SUVmean was 0.965 ± 0.059 (ratio 1.62 ± 0.99).

-Pathological peri-implant tissues

Hypermetabolism was observed in three patients. These three patients had multiple implants, some of which were metabolically normal and the others in the same arcade were pathological. This situation enabled qualitative and quantitative comparison of the normal and pathological implants.

•Patient 1

**Figure 1:**
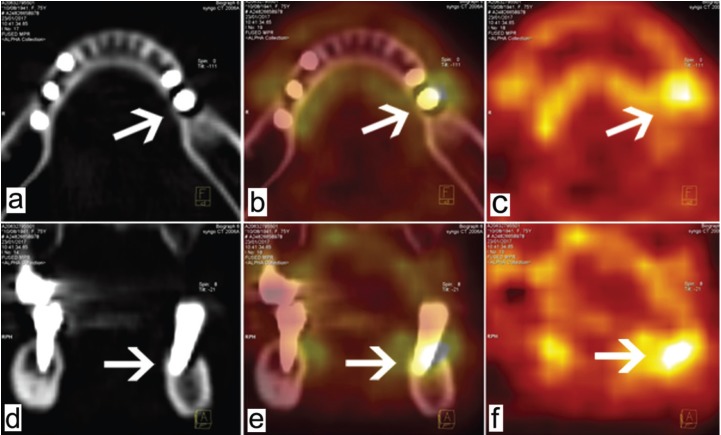
Patient 1. Reconstructed FDG PET/CT images acquired parallel to the occlusal plane (a, b, c) and parafrontally (d, e, f). Position 36 (arrows) presents peri-implant hypermetabolism and the other implant sites are normal.

This 75-year-old woman underwent FDG PET/CT imaging for lymphoma follow up. She had five mandibular implants (at positions 35, 36, 44, 45, and 46) that had been inserted 5 years previously. Position 36 showed peri-implant hypermetabolism (Fig. 1). Clinical assessment revealed peri-implantitis with mobility of this implant; the other four implants were normal. An orthopantomogram showed terminal resorption around the implant at position 36. This implant was removed a few days after the FDG PET/CT examination due to terminal mobility.

•Patient 2

**Figure 2:**
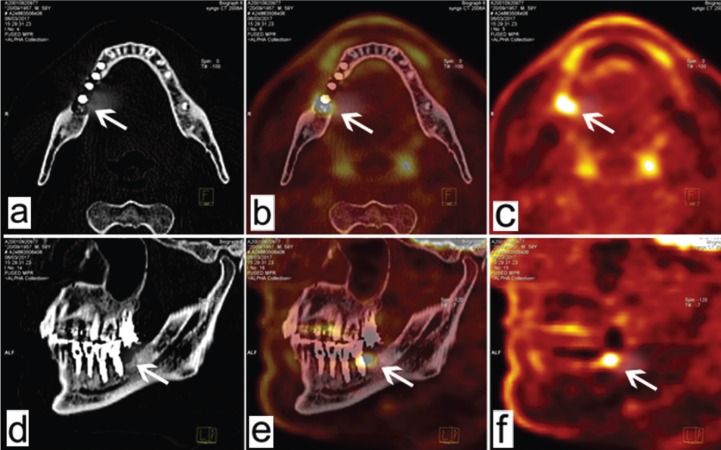
Patient 2. Reconstructed FDG PET/CT images acquired parallel to the occlusal plane (a, b, c) and parasagittally (d, e, f). Position 47 (arrows) shows clinical peri-implant hypermetabolism, whereas the other implant sites are normal.

This 59-year-old man had received six implants (at positions 24, 26, 44, 45, 46, and 47) 1 year previously. Position 47 showed peri-implant hypermetabolism, and normal peri-implant metabolism was observed at all other positions (Fig. 2). Clinically, position 47 had a distal probing depth of 5 mm with bleeding. This implant had been placed in conjunction with the insertion of a bone graft substitute in the distal position (beta-tricalcium phosphate). Surgical curettage by access flap was performed 10 days after the FDG PET/CT examination to remove an inflammatory granuloma distal to implant 47.

•Patient 3

**Figure 3:**
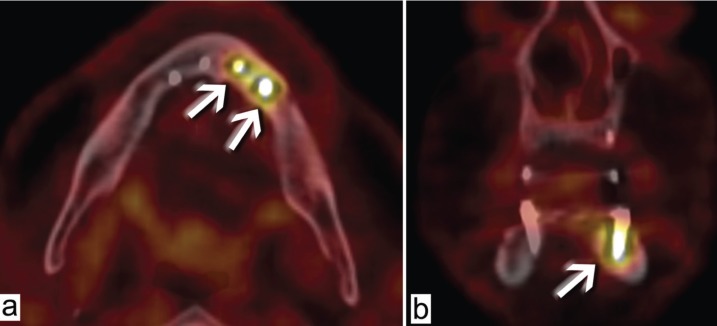
Patient 3. FDG PET/CT fusion images in horizontal (a) and frontal (b) sections. Positions 31 and 33 (arrows) show peri-implant hypermetabolism, whereas positions 41 and 43 are metabolically normal.

This 73-year-old man underwent FDG PET/CT imaging for follow up of pulmonary neoplasia in remission. The patient had received four mandibular implants (at positions 31, 33, 41, and 43) 10 years previously as part of an adjacent implant-stabilized complete prosthesis. Positions 31 and 33 showed peri-implant hypermetabolism, whereas positions 41 and 43 seemed to be metabolically normal (Fig. 3). During clinical examination, the patient explained that he had refused oral follow up since prosthesis placement, against the advice of his physician. Clinical assessment showed probing depths of 8–10 mm with bleeding around the implants at positions 31 and 33, whereas positions 41 and 43 had probing depths of 3–4 mm without bleeding.

In this series, all patients with peri-implantitis also had normal implants in the same arcade. The peri-implant SUVs of sites with peri-implantitis (mean, 7.35 ± 1.73) were significantly greater than those of healthy implant sites (mean 1.99 ± 0.62; *p* < 0.001).

## Discussion

In order to assess the interests of functional imaging in the field of implantology, this study examines FDG PET/CT images of dental implants.

-Study limitations

This observational study was conducted with unselected patients who comprised an unbiased sample of the population undergoing FDG PET/CT imaging. However, the majority of these patients underwent examinations for the staging of neoplastic pathologies, which introduces selection bias into any extrapolation of the study observations to a population of patients with implants, but no other comorbidity.

To minimize radiation exposure, low-dose CT examinations were performed; this methodology reduces image accuracy compared with classic CT and CBCT, which are commonly indicated for the planning of oral implant treatment. Thus, the spatial resolution of the images used for this study was suboptimal (12). FDG PET/CT equipment can generate higher-resolution CT images, albeit at the cost of increased X-ray exposure (13).

FDG PET/CT imaging involves “double radiation” by FDG PET (injection of 2.5 MBq/kg) and CT scan (absorption of X-rays). Consequently, the effective radiation dose ranged from 10 to 20 mSv. The cumulative radiation dose of FDG PET/CT is generally equivalent to that of standard CT. In oral imaging, a retroalveolar X-ray delivers 4 µSv, an orthopantomogram delivers 20 µSv, and a CBCT delivers 100–300 µSv radiation (14). The exposure to radiation derived from whole-body FDG PET/CT scan is significantly greater than that generated by conventional oral imaging, but not greater than that resulting from conventional radiological exams. FDG PET/CT examination of the oral cavity alone would generate less radiation while improving the CT resolution.

-Relevance of the study

This study provides FDG PET/CT data on implants (15). Thus, the relevance of this study is twofold: it provides the potential for future prospects in implantology, and it contributes to better analysis of oral cavity images generated in nuclear medicine.

In our sample, healthy implant sites with normal osteointegration showed normal metabolic activity, similar to that of normal dental and periodontal tissue. All observed hypermetabolism was associated with active peri-implantitis.

Indeed, it paves the way for better interpretation of peri-implant images by nuclear physicians, who routinely interpret scans targeting extra-oral indications (16). The descriptions provided herein constitute a useful first step analysis for the interpretation of these images by nuclear physicians (17).

Our study shows the potential role of functional imaging in the diagnosis and monitoring of peri-implantitis in clinical practice. Additionally, FDG PET/CT imaging can be useful for the identification of therapeutic targets. Even though the resolution is suboptimal, the clarity of these images allows easy communication among physicians and between physicians and patients.

From an experimental point of view, FDG PET/CT imaging may represent a new tool for the understanding of implant physiology and the study of peri-implant pathologies (18). This methodology could also be used to evaluate the treatment of peri-implantitis by assessing the level of metabolic activity in response to therapy (19). Many treatment protocols have been described in the literature, making comparative evaluation difficult (20-24).

This work represents a first step and identifies several directions for additional research. Further studies should involve statistical analysis to determine a threshold of positivity for the diagnosis of peri-implantitis and to establish values of sensitivity and specificity. Finally, this study provides a gateway for the use of FDG-PET/CT imaging for the assessment of other pathologies of the oral cavity, such as inflammatory periodontal lesions, radio-induced and chemo-induced osteonecrosis of the maxilla, and maxillary cysts and tumors.

## Conclusions

The purpose of this work was to determine whether FDG-PET/CT imaging is of relevance in implantology. The collection of information on the implants of patients who have undergone FDG-PET/CT imaging has helped to lay a foundation for implant analysis through FDG-PET/CT.

This study demonstrated that an implant showing normal osteointegration presents metabolic activity similar to that of the surrounding healthy bone and periodontium. Peri-implant hypermetabolism is pathological and should lead to the suspicion of peri-implantitis.

This work highlights the considerable potential of FDG PET/CT imaging in implantology. This methodology is relevant not only in the clinical setting in the context of peri-implantitis, but also because it paves the way for the development of novel research tools.
